# The Effects of Nordic Walking With Poles With an Integrated Resistance Shock Absorber on Cognitive Abilities and Cardiopulmonary Efficiency in Postmenopausal Women

**DOI:** 10.3389/fnagi.2020.586286

**Published:** 2020-10-19

**Authors:** Katarzyna Domaszewska, Magdalena Koper, Krystian Wochna, Urszula Czerniak, Katarzyna Marciniak, Maciej Wilski, Dorota Bukowska

**Affiliations:** ^1^Department of Physiology and Biochemistry, Poznań University of Physical Education, Poznań, Poland; ^2^Department of Adapted Physical Activity, Poznań University of Physical Education, Poznań, Poland; ^3^Laboratory of Swimming and Water Lifesaving, Poznań University of Physical Education, Poznań, Poland; ^4^Department of Anthropology and Biometry, Poznań University of Physical Education, Poznań, Poland; ^5^Department of Physical Activity Sciences and Health Promotion, Poznań University of Physical Education, Poznań, Poland; ^6^Department of Neurobiology, Poznań University of Physical Education, Poznań, Poland

**Keywords:** walking training, neurotrophins, maximal oxygen uptake, Beck Depression Inventory, Stroop test

## Abstract

Late adulthood is associated with atrophy of brain areas, which contribute to cognitive deterioration and increase the risk of depression. On the other hand, aerobic exercise can improve learning and memory function, ameliorate mood, and prevent neurodegenerative changes. This study demonstrates the effect of Nordic walking (NW) and NW with poles with an integrated resistance shock absorber (NW with RSA) on aerobic capacity and body composition in postmenopausal women. It also measures the brain-derived neurotrophic factor (BDNF) and glial cell line-derived neurotrophic factor (GDNF) serum levels and determines correlations with cognitive functions and depression symptoms. These relationships with the use of NW with RSA as a new form of exercise have not been described thus far. In this study, 31 women (NW – 16, NW with RSA – 15) participated in eight weeks of training. The findings showed that only NW with RSA training caused a significant decrease in body mass and body mass index (*p* < 0.05). There were no significant changes in GDNF levels between groups studied. Regarding BDNF, a significant decrease (*p* < 0.05) in the NW group and an increase (not statistically significant) in the NW with RSA group was found. A comparative analysis of cognitive and depression outcomes and changes in BDNF and GDNF concentration showed no significant differences in the efficacy of either form of training. Training loads resulted in a significant increase in VO_2_max in both the NW (*p* < 0.01) and NW with RSA (*p* < 0.05) groups. This indicates an improvement in cardiopulmonary efficiency of the examined women.

## Introduction

In the aging process, a series of changes occur in the structures and systems of the human body. Functional changes associated with atrophy of certain areas of the brain, especially the hippocampus and prefrontal and temporal cortices, can lead to age-related cognitive decline (Raz et al., 2005; Driscoll et al., 2009; Kennedy et al., 2009), risk of depression (Steffens et al., 2000; O’Shea et al., 2018; Szymkowicz et al., 2019) and neurodegenerative diseases (Wilson et al., 2002). There is evidence to suggest that these changes are correlated with reduced levels of brain-derived neurotrophic factor (BDNF) (Tapia-Arancibia et al., 2008; Erickson et al., 2012) and glial cell line-derived neurotrophic factor (GDNF) (Wang et al., 2011; Sharma et al., 2016; Tsybko et al., 2017) in older adults and rodents. BDNF and GDNF belong to the family of trophic factors identified in the nervous system (Siamilis et al., 2009; Ghanbarzadeh et al., 2016) and various non-neuronal tissues, including skeletal muscles (Dupont-Versteegden et al., 2004), especially in response to physical exercise. BDNF regulates synaptogenesis and survival of adult neurons, enhances the mechanism of synaptic plasticity, thereby influencing cognition, and preventing depression and Alzheimer’s disease (Weinstein et al., 2014; Ferrer et al., 2019; Ng et al., 2019). The main function of GDNF is to exert a protective effect on dopaminergic and cortical neurons, and spinal motoneurons, consequently improving motor function and thus preventing Parkinson’s disease (Zigmond et al., 2009; Lau et al., 2011; McCullough et al., 2013). The important contribution of GDNF in the pathophysiology of neuropsychiatric disorders is also reported (Chu et al., 2018).

Human and animal studies have shown that physical activity can stimulate synthesis and the release of endogenous neurotrophins. The number of publications concerning the beneficial effect of various types of aerobic exercise and training on enhancing BDNF levels in the brain and peripheral blood both in animal models (Afzalpour et al., 2015; Eldomiaty et al., 2017; TaheriChadorneshin et al., 2017; Algaidi et al., 2019) and humans (Currie et al., 2009a; Damirchi et al., 2014; Huang et al., 2014; Dinoff et al., 2017) is constantly increasing. Training-induced increase in BDNF levels improves cognitive function (Best et al., 2015; Hákansson et al., 2017; Nilsson et al., 2020) and mood (Rethorst et al., 2009) in healthy people and people with cognitive impairment (Nascimento et al., 2015) and depression (Schuch et al., 2016; Kering et al., 2017). The animal studies confirm observations that training exerts a beneficial effect on learning and memory and ameliorates depression-like behavior (Lin et al., 2012; Marlatt et al., 2012; Lu et al., 2014).

In light of numerous BDNF studies, there is little information concerning the influence of exercise training on GDNF levels in humans (Roh and So, 2017). More extensive studies in animals, mainly rodents, provide data on GDNF levels in the hippocampus of rats exposed to stress (Jiang et al., 2014), in the striatum of hemiparkinsonian mice (Speck et al., 2019) and the spinal cord and skeletal muscles (McCullough et al., 2013; Vianney et al., 2013) in response to physical activity.

There are several lines of evidence to indicate that physical exercise not only can generally slow down aging and prevent chronic, psychiatric and neurodegenerative diseases, but is very important in maintaining normal brain function or softening its progressive loss (Haskell et al., 2007; Pedersen and Saltin, 2015; Tan et al., 2017). This issue is especially important in women because of the gender differences associated with aging, namely the consequences of menopause. A decrease in estrogen production and corresponding estrogen deficiency leads to a decline in physical functions and negatively influences cognition and mood, and contributes to the development of neurodegenerative processes (Li et al., 2014). Lack of neuroprotective action of estrogen in postmenopausal women causes their risk of Alzheimer’s disease to be higher when compared to age-matched men (Vina and Lloret, 2010).

In the above context, this investigation was undertaken to demonstrate the impact of walking training, i.e., Nordic walking (NW) with classic poles and NW with poles with an integrated resistance shock absorber (NW with RSA), on BDNF and GDNF serum levels, and correlations with cognitive functions and depression levels in postmenopausal women. The new form of NW is training with modified poles, which allows combining aerobic and strength training. These modified poles contain a built-in RSA. An elastic tape between two permanent elements in RSA poles allows additional resistance to be obtained by increasing the overall intensity of exercise and calorie consumption. Literature analysis indicates that this is the first study to describe these relationships.

## Materials and Methods

### Study Design and Participants

Initially, 50 women aged 60–75 were recruited for the study. Subjects were recruited by advertisements in local media and at informative events and were qualified to participate in the project based on medical history and cardiology tests. The following exclusion criteria have been applied (presence of at least one of the factors listed below): diseases of the locomotor system preventing independent movement, giant obesity, active or post cancerous disease (ongoing radiation or chemotherapy treatment), liver diseases (ALT > 3× borderline) except for liver disease, chronic kidney disease (eGFR < 30 mL/1.73 m^2^/min), acute inflammation (CRP > 5 mg/dL), unstable ischemic heart disease, after an ischemic or hemorrhagic stroke (<6 months), post-STEMI (ST-elevation myocardial infarction) women with a drug-eluting stent implantation, NSTEMI (non-ST-elevation myocardial infarction) (<12 months), inherited metabolic disorders (phenylketonuria and galactosamia), autoimmune diseases (an acute thyroiditis, celiac disease, systemic connective tissue disease, hemolytic anemia, vitiligo, Addison’s disease, hyperbilirubinemia), non-specific enteritis (Crohn’s disease and ulcerative colitis), psychological disorders, antibiotic therapy, steroid therapy (ongoing), drug, and alcohol addiction (a daily consumption of more than one portion of alcohol). Finally, 40 women (mean age 66.64 years, SD = 4.20) were included in the project and were randomly assigned to two groups with a different training program. Randomization was performed as simple random allocation; each subject identifier was forwarded to a person who was not involved with the conduct of the study, and who performed the randomization blindly using a computer list. All subjects were of Caucasian origin, in particular, a native Polish population from the Great Poland region. The subjects were asked not to change their eating habits for the duration of the project and not to perform any additional physical activity, except for the one carried out in the research project. Five women withdrew from the project, while four were excluded due to performing additional physical activity. Thus, the results of 31 people (NW – 16, NW with RSA – 15) were statistically analyzed. An information sheet was provided to each woman approached to participate in the study and on agreement to participate, informed written consent was obtained. The entire study was conducted over nine weeks (from the 13th of February 2019 until the 17th of April 2019).

### Training Program

The research experiment was held twice a week for 8 weeks (16 training sessions). Training for both groups was performed at the same time. Women from the NW group used classic poles, while the NW with RSA group used poles with an integrated RSA having an elastic resistance of 4 kg (Slimline Bungy Pump, Sports Progress International AB, Sweden). Each training session started with 10–15 min of warm-up. After each half of the planned distance (about 1.7–2.2 km at a rate of about 1 km per 10 min) participants of the classes performed strength exercises and balance training (15 min). After the rest of the planned distance, stretching exercises took place at the end of the training (15 min). During the meetings, the walking distance from 3.5 to 4.5 km, as well as the number of exercises performed from 8 to 12 repetitions was gradually increased. Exercise intensity corresponded to 50% HRR (heart rate recovery) during 1–8 training sessions, while from sessions 9–16 the intensity increased to 65–70% HRR and was monitored based on heart rate (HR) (Polar Electro Oy, Kernpele, Finland) (Marciniak et al., 2020). The minimum required presence at 13 training sessions was adopted (80%). The trainer conducting the classes had appropriate qualifications. The training plan included American College of Sports Medicine (ACSM) recommendations for adults and healthy older people: one set of 8–10 exercises for major muscle groups 2–3 days per week; most exercisers should perform 8–12 repetitions of each exercise.

### Blood Analysis

Testing sessions were scheduled 4 days before the training period and 4 days after the final training bout. Subjects were instructed to abstain from physical exercise between the final training bout and the final test. Participants reported to the laboratory between 07.00 and 8.00 h after an overnight fast. The time of day was held constant for every subject (±30 min). Blood samples (10 mL) were taken from the ulnar vein after a resting period of at least 10 min using a S-Monovette syringe tube (SARSTEDT, Germany), then placed in tubes containing a clot activator, and centrifuged (1500 × *g*, 4°C, 4 min) (Universal 320R; Hettich Lab Technology, Tuttlingen, Germany) to separate the serum. The samples were frozen and stored at −80°C until the time the analyses were performed (U410, Ultra-Low Temperature Freezer, New Brunswick Scientific, United States).

Serum BDNF levels were quantified using a commercially available ELISA (enzyme-linked immunosorbent assay) kit (Shanghai Sunred Biological Technology Co., Ltd.). The concentrations of GDNF were measured with an ELISA kit (Cloud-Clone Corp., Katy, TX, United States). The sensitivities of the ELISA kits were as follows: 0.05 and 0.058 ng/mL, respectively. The intra- and inter-assay coefficients of variation (CVs) were less than 10 and 12%, respectively. The samples were read using a Synergy 2 SIAFRT multi-detection microplate reader (BioTek, United States) at the manufacturer’s recommended wavelength.

### Exercise Test

Aerobic capacity was assessed with the modified Astrand–Rhyming protocol for predicting VO_2_max by an ergometer Kettler DX1 Pro, (Ense – Parsit, Germany) and HR was monitored using a Polar A-5 pulse meter (Polar Electro Oy, Kernpele, Finland) (Gillett, 1993). The predicted VO_2_max was read from the nomogram (Astrand and Ryhming, 1954) or accompanying tables (Astrand and Ryhming, 1954) and multiplied by both the Astrand and the von Dobeln age correction factors. These two predictions in L/min were then converted to mL/kg/min.

### Body Composition

Bioelectric impedance analysis (BIA) is commonly used in field surveys and also as a supplement to conventional anthropometry (Heyward and Wagner, 2004). The body composition of women was estimated with BIA using the TANITA MC-980MA (TANITA, Japan), following the directions and procedures of the manufacturer. The unit provides a profile for the individual including weight, % fat, % fat-free mass, muscle mass, and visceral fat tissue. The women stood erect holding the hand electrodes and with bare feet on the contact electrodes of the BIA unit. Waist circumference was measured halfway between the lower rib and the iliac crest in a horizontal plane. Hip circumference was measured as the widest horizontal circumference over the buttocks.

### Psychological Examination

Depressive symptoms were assessed with the Beck Depression Inventory (BDI). The BDI is a self-reported measure of depression consisting of 21 items covered to detect the total score for measuring the symptoms of depression (Beck et al., 1988). Each item is scored with 0–3 points. The items are summed and the total possible score for the scale ranges from 0 to 63, with higher values indicating greater depression levels. It has proven to be sensitive to exercise-induced changes in healthy adults and postmenopausal women without depression (Bernard et al., 2015).

The Stroop Color and Word Interference Test was developed to measure selective attention and cognitive flexibility (Stroop, 1935). It refers to the impairment of the reading speed and color recognition due to interfering information and consists of two parts. In the first part, Reading Color Names in Black (RCNb), the subject is required to read 10 rows of five words for color names written in black on a white sheet as soon as possible. In the second part, Naming Color of Word/different (NCWd), subjects should name the individual font color as soon as possible, where the font color of the word is different from the color that is written. Change of reaction form is required (switching from content to color). The time in seconds needed to make the first and second parts is estimated (Homack and Riccio, 2004).

The Trail Making Test (TMT) is a widely used neuropsychological test of visual attention and task switching (Salthouse, 2011). It consists of two parts in which the subject is instructed to connect a set of 25 dots as quickly as possible while still maintaining accuracy. If the subject makes an error, the test administrator corrects them before the subject moves on to the next dot (Bowie and Harvey, 2006). In the first part, the targets are all numbers (1, 2, 3, etc.) and the test taker needs to connect them in sequential order as fast as possible. The first part is used primarily to examine cognitive processing speed. In the second part, the participant is to draw lines to connect circled numbers and letters in an alternating numeric and alphabetic sequence (i.e., 1-A-2-B, etc.) as fast as possible. This part is used to examine executive functioning (Tombaugh, 2004; Bowie and Harvey, 2006). The goal of the test is to finish both parts as quickly as possible, with the time taken to complete the test being used as the primary performance metric.

The Verbal Fluency Test (VFT) is one of the best known and useful neuropsychological tools based on the language functions diagnosis. VFT allows one to discover disorders within the cognitive sphere, including evaluating many functions such as verbal fluency, processes of attention, information processing rate, working memory, and executive functions (Ruff et al., 1997). There are two versions of VFT: using the formal (letter-related) criterion (i.e., phonemic fluency) and semantic (category-related) criterion (i.e., semantic fluency). In the formal version of VFT the respondent generates the highest possible number of words beginning with a given letter. The semantic fluency test consists of listing, by the examiner, the highest possible number of words belonging to a certain category (in our case “fruit”). We used the letter “P.” Each task is performed within 1 min (Ponichtera-Kasprzykowska and Sobów, 2014).

### Statistical Analyses

The obtained results were analyzed statistically using the Dell Statistica data analysis software system (version 13, software.dell.com, Dell Inc., Round Rock, TX, United States). The sample size was calculated based on data from study of Zoladz et al. (2014) that determined the average level of plasma BDNF. In addition, this study shows methodological similarities with the present study (the 8-weeks NW training and age). After calculations adopting a power as 1-beta error probability: 95%, effect size: 0.80, and error assumed as alpha: 0.03 (two-sided), eight participants were assigned to allocate equally into each group (four NW and four NW with RSA). Therefore, the present study was initiated with 40 women divided randomly between the NW (*n* = 20) and NW with RSA (*n* = 20) groups. The data are presented as mean and standard deviation (SD). The normality of distributions was verified using the Shapiro–Wilk test. It was assumed that the analysis of repeated-measures ANOVA 2 × 2 (time × group) will be performed if the differences in variables over time and between groups are significant. The Mann–Whitney U test and the Wilcoxon test were employed for non-normally distributed variables, respectively, to evaluate the significance of differences between the groups and test dates. Spearman’s rank analysis was used to calculate correlation coefficients. The level of statistical significance was set at *p* ≤ 0.05. Effect sizes (ESs) were calculated as the difference between means divided by the pooled SD. Using Cohen’s (1988) criteria, an effect size ≥0.20 and <0.50 was considered small, ≥0.50 and <0.80 medium, and ≥0.80 large.

## Results

Comparative analysis of somatic characteristics, cardiopulmonary efficiency, and blood BDNF and GDNF levels measured before and after an 8-week NW and NW with RSA training program is presented in Table 1. The 8-week training program caused a significant decrease in waist circumference of subjects in both NW [*p* < 0.05 (ES: 0.13)] and NW with RSA [*p* < 0.01 (ES: 0.27)] training groups. There was no significant difference in post-workout change in waist circumference between the two groups. NW with RSA training caused a significant decrease in body mass [*p* < 0.05 (ES: 0.09)] and BMI [*p* < 0.05 (ES: 0.10)]. Statistical analysis showed a significant difference in the change of the described indicators between the examined groups. Training loads implemented in the project resulted in a significant increase in cardiorespiratory efficiency in the NW [*p* < 0.01 (ES: 0.93)] and NW with RSA [*p* < 0.05 (ES: 0.54)] groups. There was no significant difference in post-workout change in cardiorespiratory efficiency between the two groups.

**TABLE 1:** Somatic characteristics, cardiopulmonary efficiency, and biochemical indices of women subjected to an 8-week NW and NW with RSA training program.

	**NW (*n* = 16) (M ± SD)**			**NW with RSA (*n* = 15) (M ± SD)**		
	**Baseline**	**9 weeks**	**Change**	**Baseline**	**9 weeks**	**Change**
Age, years	66.38 ± 3.74			68.00 ± 4.33		
Body height, cm	159.80 ± 5.09			161.91 ± 3.62		
Body mass, kg	67.70 ± 9.99	67.59 ± 10.21	−0.11 ± 0.75	73.77 ± 10.47	72.81 ± 10.14*^a^	−0.97 ± 1.38^#*a*^
BMI, kg/m^2^	26.44 ± 3.02	26.39 ± 3.03	−0.07 ± 0.31	28.16 ± 3.95	27.78 ± 3.76*^a^	−0.38 ± 0.54^#*a*^
FAT, %	35.09 ± 4.37	35.32 ± 3.88	0.16 ± 1.43	37.75 ± 4.84	37.52 ± 4.94	−0.23 ± 1.21
FFM, %	43.19 ± 5.29	43.21 ± 5.73	0.04 ± 1.17	45.47 ± 4.96	45.05 ± 4.14	−0.42 ± 1.14
SMM, kg	40.97 ± 5.02	41.01 ± 5.47	0.06 ± 1.12	43.15 ± 4.25	42.77 ± 3.92	−0.38 ± 1.09
VFA	9.07 ± 1.53	9.07 ± 1.53	0.00 ± 0.00	10.15 ± 1.86	10.08 ± 2.06	−0.08 ± 0.64
Waist circumference, cm	83.10 ± 8.63	82.02 ± 8.19*^a^	−1.08 ± 1.79	88.50 ± 9.88	85.93 ± 9.19*^b^	2.57 ± 3.05
Hip circumference, cm	99.44 ± 7.00	99.31 ± 6.93	−0.13 ± 9.93	104.43 ± 5.81	104.00 ± 6.31	−0.43 ± 2.69
VO_2_max, mL/kg/min	27.68 ± 4.89	32.14 ± 4.79*^b^	4.45 ± 4.38	28.05 ± 4.45	30.45 ± 4.49*^a^	2.40 ± 3.87
BDNF, ng/mL	9.29 ± 15.48	7.76 ± 13.68*^a^	−1.53 ± 5.04	9.52 ± 11.44	9.78 ± 12.79	0.26 ± 5.36
GDNF, ng/mL	1.69 ± 0.88	1.86 ± 0.85	0.17 ± 0.76	1.67 ± 1.00	1.85 ± 1.13	0.18 ± 0.68

Values are means (± SD). BMI, body mass index; FAT, body fat mass; FFM, body free fat mass; SMM, skeletal muscle mass; VFA, visceral fat area; VO_2_max, maximal oxygen uptake; BDNF, brain-derived neurotrophic factor; GDNF, glial cell line-derived neurotrophic factor. ^*a*^*p* < 0.05. ^*b*^*p* < 0.01. *Level of significance of change between baseline and after nine weeks for each group ^#^Level of significance of post-workout changes in the studied indicators between the examined groups.

In both NW and NW with RSA groups, a slight but not significant increase in blood GDNF concentrations was revealed. However, there were no significant changes in blood GDNF levels between groups studied. In the group covered by traditional NW training, a significant decrease in BDNF blood concentration was found [*p* < 0.05 (ES: 0.11)]. The opposite was noted in the NW with RSA group. However, this change was not statistically significant.

A comparative analysis of cognitive and mental health outcomes in response to the intervention program is shown in Table 2. The 16 training sessions of NW caused no significant changes in the cognitive and mental health outcomes. In the case of variables such as depression, naming interference, and executive function, expected positive changes were observed, but they were not statistically significant. Similarly, under the influence of NW with RSA training no significant changes were noted in most of the tested parameters. However, in the naming interference, statistically significant positive changes as an effect of the applied exercise program were noted [*p* < 0.05 (ES: 0.55)]. Statistical analysis indicated that no significant differences in post-workout changes results were present between the two groups studied (NW and NW with RSA).

**TABLE 2:** Cognitive and mental health outcomes in response to the intervention program.

	**NW (*n* = 16) (M ± SD)**			**NW with RSA (*n* = 15) (M ± SD)**		
	**Baseline**	**9 weeks**	**Change**	**Baseline**	**9 weeks**	**Change**
BDI (points)	9.1 ± 8.27	8.3 ± 6.74	−0.8 ± 4.18	8.33 ± 7.02	7.2 ± 6.26	−1.1 ± 5.18
STROOP 1 (s)	26.1 ± 2.46	26 ± 2.89	−0.1 ± 2.35	24.8 ± 3.99	24.9 ± 3.78	0.14 ± 2.39
STROOP 2 (s)	68.7 ± 11.78	65.6 ± 13.43	−3.1 ± 9.45	69.9 ± 13.63	62.9 ± 11.54*^a^	−7.0 ± 9.79
TMT A (s)	35.9 ± 10.77	33.8 ± 9.7	−2 ± 9.55	34.0 ± 4.31	37.0 ± 10.54	2.97 ± 9.44
TMT B (s)	84.4 ± 31.84	77.3 ± 29.6	−7.2 ± 27.52	94.8 ± 39.71	92.1 ± 48.59	−2.7 ± 23.13
VF L (words)	17.7 ± 6.38	16.9 ± 4.49	−0.8 ± 4.96	15.1 ± 4.61	14.8 ± 6.25	−0.2 ± 3.61
VF S (words)	14.7 ± 2.44	15.4 ± 2.03	0.7 ± 3	14.9 ± 3.03	15.4 ± 3.64	0.53 ± 2.83

Values are means (±SD). BDI, Beck’s Depression Inventory; STROOP 1, reading interference; STROOP 2, naming interference; TMT A, cognitive processing speed; TMT B, executive function; VF L, formal fluency (letter-related criterion); VF S, semantic fluency (category-related criterion). ^*a*^*p* < 0.05. *Level of significance of the change between baseline and after 9 weeks for each group.

The findings obtained in this study indicate some relationships between variable changes before and after an 8-week training program. However, at the beginning it should be emphasized that no correlation was found between post-workout changes in cardiopulmonary efficiency and changes in BDNF and GDNF blood levels in either of the NW and NW with RSA groups.

Significant correlations between BDNF concentrations and certain cognitive and mental health outcomes were noted among respondents from both study groups (NW and NW with RSA). In the NW group, higher BDNF concentrations resulted in better early TMT B results (*r* = −0.51; *p* = 0.045). In addition, changes in BDNF levels correlated with changes in TMT B results (executive function) in this group (*r* = −0.67; *p* = 0.004). Moreover, women with higher GDNF levels at baseline, achieved better results in the VF S test (*r* = 0.61; *p* = 0.012). We observed correlations between GDNF concentrations and VF L test results after the NW training program (*r* = −0.54; *p* = 0.032). Furthermore, changes in GDNF levels positively correlated with changes in VF S (*r* = 0.77; *p* = 0.001). There was also a negative correlation between the post-workout change in body mass, BMI, FFM, SMM, and the change in resting GDNF concentrations in the blood (*p* < 0.05). In the NW with RSA group, the higher the BDNF concentration, the better the TMT A test results were at baseline (*r* = −0.55; *p* = 0.034). BDNF concentrations were also found to correlate significantly with the results from the VF L test both at the beginning (*r* = 0.56; *p* = 0.031) and at the end of experiment (*r* = 0.65; *p* = 0.008). Additionally, a correlation between BDNF concentration and VF S test results was also recognized in the NW with RSA group (*r* = 0.70; *p* = 0.004) after the experiment was completed.

Statistical analysis showed that there were no significant correlations between GDNF concentrations and the results obtained in the field of cognitive and mental health in the NW with RSA group. Moreover, in the group of women included in the NW with RSA training program, a significant relationship was observed regarding the post-workout change in waist circumference and change in BDNF concentration (*r* = −0.71; *p* = 0.003) in addition to GDNF concentration (*r* = 0.61; *p* = 0.015). Also, analysis of post-workout changes in the levels of cardiopulmonary efficiency showed correlations with the changes in TMT A (*r* = −0.53; *p* < 0.05).

## Discussion

In this study in postmenopausal women, BDNF and GDNF concentrations in the blood were investigated along with cognitive functions and depressive symptoms to evaluate the effects of two types of walking training, i.e., NW with classic poles and NW with RSA using poles equipped with a system that provides resistance whenever the pole is pressed down. Relationships with cardiopulmonary efficiency and body composition were also analyzed. The main result presented herein is that NW compared to NW with RSA is a more effective form of walking training for postmenopausal women.

There is a lot of research on the effectiveness of the NW activity in reducing body weight, improving cardiopulmonary efficiency, glucose, and lipid metabolism in postmenopausal women (Latosik et al., 2014; Hagner-Derengowska et al., 2015). It has also been shown to have a beneficial effect on improving cognitive function, mental health, and slowing down neurodegenerative changes associated with aging (Gmiąt et al., 2018).

Regarding NW with RSA, there are currently no published scientific reports on the effects of this relatively new kind of training on neurotrophins level, cognitive function, and depressive symptoms in people of all ages. However, there is one available study concerning the effects of the NW with RSA training on the functional fitness of older women. The authors indicated that in comparison to NW, the NW with RSABP training provides additional resistance effort during marching, improved muscle strength, and aerobic endurance as a result of increased muscle activation (Marciniak et al., 2020). According to information from the NW with RSA producer, the use of NW with RSA poles and NW poles provides an average of 21 and 18% higher oxygen consumption, respectively, compared to normal walking^1^. Thus, the intensity of NW with RSA training is higher causing it to have a greater impact on the body.

A comparative analysis of both forms of walking used in the present study revealed that 16 training sessions of NW and NW with RSA did not cause significant changes in the cognitive and mental health outcomes. For variables such as depression, naming interference, and executive function, expected positive changes were observed, but they were not significant. Statistical analysis showed that between NW and NW with RSA groups there were no differences in the scope of post-workout changes of examined indicators.

Many studies have been done to identify appropriate forms of exercise and training that prevent the decline of cognitive function (Lista and Sorrentino, 2010) and reduce the risk of depression (Yuenyongchaiwat et al., 2018) during aging. In response to physical activity, there is an increase in BDNF levels in the brain and peripheral blood (Kang et al., 2020). For GDNF, the available data in humans are scarce (Roh and So, 2017), and not many arise from animal studies (McCullough et al., 2013; Vianney et al., 2013; Jiang et al., 2014; Speck et al., 2019).

A meta-analysis based on 55 studies showed that 39% of them documented a significant increase in BDNF blood levels after acute exercise, and 61% of studies reported no post-workout change. Longer training units, regardless of the type of exercise performed, were more effective. Gender and baseline levels of physical fitness had a significant impact on post-workout BDNF changes (Dinoff et al., 2017).

On the other hand, there is evidence of a post-workout decrease in BDNF concentrations in response to diverse training programs (Correia et al., 2010; Walentukiewicz et al., 2018). In our study concerning post-workout changes in BDNF blood levels in the women examined, the different effects of training loads were demonstrated. In subjects from the NW group, there was a significant decrease in BDNF concentrations after 8 weeks of training. This finding corresponds well with that received after 12 weeks of NW training in elderly women (Walentukiewicz et al., 2018). Unlike the NW group, the upward trend in BDNF concentrations in the NW with RSA group observed in our study is difficult to explain, especially since no reports of this type of training are available.

Approximately three-quarters of BDNF production both at rest and during exercise occurs in the brain. Its decreased concentration in the periphery may result from greater use by skeletal muscle to regulate lipid metabolism in muscle fibers and absorption by the hippocampus and prefrontal cortex, which is pivotal for cognitive abilities (Rasmussen et al., 2009). Animal studies have shown that exercise causes an increase of BDNF uptake from the blood which is then involved in regeneration processes (Yu et al., 2017).

It should be noted that neither NW nor NW with RSA training used in the project caused an increase of GDNF concentration in the blood. Due to the lack of research on the impact of these forms of physical activity on GDNF concentration, the obtained results cannot be confirmed. However, there is a study on the effects of pharmacological treatment on GDNF levels in women with schizophrenia and depression (Skibinska et al., 2017).

It is well known that high levels of endogenous neurotrophins, BDNF and GDNF, in the brain and peripheral blood reduce the hippocampus gray matter loss, improve memory, and reduce the occurrence of depression in the elderly (see section “Introduction”). The direction and size of changes in the concentration of neurotrophins is related to the duration of the training, its intensity, the type of exercises performed, and the subjects’ health. Erickson et al. (2012) demonstrated the high effectiveness of moderate-intensity aerobic exercises performed by older people on the increase in BDNF concentration, hippocampus volume, and cardiorespiratory fitness compared to stretching exercises. Physical activity varying in intensity did not reveal changes in post-workout BDNF plasma concentrations in the elderly. Despite this, there was an observed increase in gray matter volume in the prefrontal cortex and beneficial effects on memory function independently of training intensity (Ruscheweyh et al., 2011). Exercises may also induce normalization of decreased BDNF serum concentration in elderly women with depression (Laske et al., 2010) and improve cognitive function and cardiorespiratory fitness in older adults with glucose metabolism disorders (Baker et al., 2010).

The NW is characterized by lower intensity of effort compared to NW with RSA. The metabolism of working muscles is dominated by a greater share of oxygen metabolism compared to NW with RSA walking at the same speed. The post-workout increase in cellular mitochondrial transformations is one of the factors responsible for increasing the VO_2_max value. Our findings indicate an 86% greater increase in the VO_2_max in women from the NW group (large effect size) in comparison to the NW with RSA group (medium effect size). The VO_2_max value is largely determined by the cardiovascular, respiratory, and metabolic fitness of the muscle, and its post-workout growth is mainly caused by moderate-intensity efforts. High-intensity training not only does not increase the VO_2_max value but is not suitable for older people (Coelho et al., 2013). The NW training leads to a decrease in body weight, body fat mass and BMI, increasing the level of aerobic capacity in people of all ages. The increase in maximum oxygen uptake results from an improvement in cardiovascular and respiratory efficiency, but also from an increase in the oxidative capacity of the muscles themselves. The improvement in aerobic capacity resulting from exercise training may be primarily a protective factor for the aging cardiovascular system and may positively affect the quality of life of the elderly (Figard-Fabre et al., 2010; Gram et al., 2010). On the other hand, the use of additional weights on the hands during NW training in the elderly does not increase energy expenditure, but only increases the activity of the muscles themselves. Due to the lack of physiological and biomechanical benefits, it is not recommended in the elderly (Schiffer et al., 2011). Strength training in the elderly significantly increases muscle mass and strength. Thus, it reduces difficulties in performing daily activities, increases energy expenditure and has a positive effect on body composition. Typical strength training leads to metabolic changes in the muscle and increase in its mass, without beneficial changes in the value of the VO_2_max. Only a small combination of strength training with endurance training increases the cardiopulmonary efficiency without weakening the post-training increase in strength and muscle mass (Cadore et al., 2014). Studies conducted on young people show that exercises performed with an intensity of 70% VO_2_max cause a greater increase in BDNF concentration than those with an intensity of 40% VO_2_max (Kim and Kim, 2018). In turn, Currie et al. (2009b) revealed a negative correlation between BDNF concentration at rest and VO_2_max value (*r* = −0.35; *p* < 0.05), and the level of physical activity (*r* = −0.43; *p* < 0.01). These results indicate that increased levels of cardiorespiratory fitness and high levels of physical activity are associated with lower concentrations of resting BDNF in the serum of healthy people (Currie et al., 2009b).

In our study, both NW and NW with RSA training lead to the improvement or maintenance of cognitive abilities and mental health as well as an increase in cardiopulmonary efficiency so immensely important to elderly people. However, with NW with RSA training, despite the greater effectiveness in reducing body weight, BMI, or waist circumference, the second period of the study showed a tendency to decrease muscle mass. Post-workout loss of muscle component may indicate the occurrence of overload changes and improper selection of training loads. Therefore, the loads applied in NW with RSA training seem to be too intense for postmenopausal women, and the classic NW seems to be more beneficial for this age group.

## Conclusion

The recent and so far, the only investigation with the use of NW with RSA training concerns the assessment of functional fitness in older women (Marciniak et al., 2020). It has been demonstrated that additional resistance during NW with RSA causes increased muscle activation and, as a result, improved muscle strength and aerobic endurance in comparison to NW. Moreover, it has a significant impact on the level of physical fitness. In our study with the use of NW with RSA and NW, a comparative analysis of cognitive and depression outcomes and changes in blood concentrations of BDNF and GDNF showed no significant differences in the efficacy of either form of training. The significant increase in VO_2_max observed in both forms of physical activity may be evidence of an improvement in cardiopulmonary efficiency. To the best of our knowledge, this is the first published report that compares the effectiveness of NW with RSA training and the traditional form of NW considering the relationships mentioned above.

Further research is needed that includes pre-menopausal women to compare the effects of NW and NW with RSA training on BDNF and GDNF levels, as well as on cognitive function, risk of depression, and cardiopulmonary efficiency. These studies would be aimed at developing effective walking training programs taking into account exercise variables such as duration, volume, intensity, and resistance used in NW with RSA in different age groups both in healthy women and women with neuropsychiatric or metabolic diseases.

## Data Availability Statement

The data analyzed in this study are available on request to the corresponding author KD, domaszewska@awf.poznan.pl.

## Ethics Statement

The studies involving human participants were reviewed and approved by the Institute for Research in Biomedicine (IRB) at the Poznań University of Medical Sciences (2019-02-07; Ethics Approval Number: 245/19). The patients/participants provided their written informed consent to participate in this study.

## Author Contributions

KD: conceptualization, methodology, writing – original draft preparation, data curation, formal analysis, resources, and project administration. MK and MW: data curation, resources, and writing – original draft preparation. KW: resources and funding acquisition. UC and KM: data curation and resources. DB writing – review and editing, data curation, resources, and funding acquisition. All authors contributed to the article and approved the submitted version.

## Conflict of Interest

The authors declare that the research was conducted in the absence of any commercial or financial relationships that could be construed as a potential conflict of interest.

## Footnotes

1 http://www.bungypump.se/
